# Digital solutions for decision support in general practice – a rapid review focused on systems developed for the universal healthcare setting in Denmark

**DOI:** 10.1186/s12875-023-02234-y

**Published:** 2023-12-14

**Authors:** Anne Clausen, Emilie Rosenfeldt Christensen, Pernille Ravn Jakobsen, Jens Søndergaard, Bo Abrahamsen, Katrine Hass Rubin

**Affiliations:** 1 OPEN – Open Patient Data Explorative Network, Odense University Hospital, Odense, Denmark; 2 The Research Unit of General Practice, Institute of Public Health, University of Southern Denmark, Odense, Denmark; 3 Department of Medicine, Holbaek Hospital, Holbaek, Denmark; 4 Research Unit OPEN, Department of Clinical Research, University of Southern, Odense, Denmark

**Keywords:** Digital solutions, Digital health, Decision support, Decision aid, General practice, Primary care

## Abstract

**Background:**

Digital health solutions hold the potential for supporting general practitioners in decision-making, and include telemedicine systems, decision support systems, patient apps, wearables, fitness trackers, etc.

**Aim:**

This review aimed to identify digital solutions developed for, tested, or implemented in general practice to support the decisions of GPs in disease detection and management, using Denmark as an example country of a universal healthcare setting.

**Methods:**

This study was conducted as a rapid review. The primary search included a database search conducted in Embase and MEDLINE. The supplementary search was conducted in Infomedia and additionally included a snowball search in reference lists and citations of key articles identified in the database search. Titles were screened by two reviewers.

**Results:**

The review included 15 studies as key articles describing a total of 13 digital solutions for decision support in general practice in Denmark. 1.123 titles were identified through the database search and 240 titles were identified through the supplementary and snowball search.

**Conclusions:**

The review identified 13 digital solutions for decision support in general practice in a Danish healthcare setting aimed at detection and/or management of cancer, COPD, type 2 diabetes, depression, liver disease or multiple lifestyle-related diseases. Implementation aspects should be reported more transparently in future publications to enable applicability of digital solutions as decision support to aid general practitioners in disease detection and management.

**Supplementary Information:**

The online version contains supplementary material available at 10.1186/s12875-023-02234-y.

## Introduction

Digital health interventions provide new approaches for utilizing health data in the prevention, diagnosis, and management of diseases [1]. Digital health covers a wide range of digital solutions such as telemedicine systems, decision support systems, patient apps, wearables and fitness trackers, etc. [2]. Digital health interventions can support healthcare providers in disease detection and prevention by providing prompts or alerts for patients at high risk of disease.

In this review, Denmark is used as an example country with universal healthcare coverage, as all citizens have access to needed medical services, which are primarily tax-funded [3]. Danish citizens have free access to a general practitioner (GP), who acts as a gatekeeper for referrals to specialist or hospital care [4], and 96% of Danes have contact with their GP over a three-year period [5]. Access to care in the secondary healthcare sector is also free, providing that the patient received a referral from their GP, ands GPs are therefore usually the first point-of-contact to the healthcare system [4]. GPs are self-employed, and general practices are funded through contracts with public authorities [4]. Practices are usually fairly small, consisting of 2–3 GPs plus nurses and secretaries, serving 1500–1800 patients per GP [4, 6].

Danish general practices are overall fully digitized, with patient records and clinical data communication between general practice, hospitals, and pharmacies fully computerized [4]. Further, digital consultations are available in general practice [4], and Denmark tops the list in an OECD comparison of European countries in eHealth adoptions [7], which overall suggests that the Danish general practice setting is quite mature in terms of digitization. The gatekeeper role that GPs occupy in the Danish healthcare system makes general practice eligible for implementation of digital health interventions to improve the early detection of patients at risk of diseases in a Danish context [4].

Digital solutions can be implemented into GP software systems as decision support systems to alert GPs of patients at high risk of disease, and aid GP decisions for referrals to diagnostic procedures or treatment initiation at specialists/the hospital. To our knowledge, there is at present no available record of digital decision support systems in general practice in Denmark. Therefore, this rapid review aimed to identify digital solutions developed for, tested, or implemented in general practice to support the decisions of GPs in disease detection and management, using Denmark as an example country of a universal healthcare setting with a fully digitized general practice sector [4, 7]. Findings from this review may be relevant to other countries with similar digitized universal healthcare systems.

## Methods

This study was conducted as a rapid review, and reported according to PRISMA guidelines where applicable [8]. The PRISMA checklist was provided in supplementary material 1.

### Data sources

The databases Embase, MEDLINE, and Infomedia were included as data sources.

### Search strategy

The search was divided into a database search and a supplementary search. The database search was performed in Embase and MEDLINE using a Boolean search strategy. The strategy consisted of three blocks: Denmark (block 1) AND General practice (block 2) AND Digital solutions for decision support (block 3). Each block consisted of keywords that were combined with the operator OR. The block search was conducted using the operator AND between the blocks. Keywords for blocks 1 and 2 were chosen after consultation with a research librarian from the library of the University of Southern Denmark. Further, keywords for block 3 were chosen from validated health app filters [9] and adapted to the current search strategy in collaboration with the research librarian. The search strategies are available in supplementary material 2.

The supplementary search was performed in Infomedia with Danish sources including the Danish Medical Journal and Dagens Medicin. The motivation for the supplementary search was to leave the search strategy open to sources that were not necessarily peer reviewed, but could still describe digital solutions relevant to the aim of this review. Infomedia was searched using the following keywords: digitale løsninger (digital solutions). Grey literature was included in the supplementary search to ensure a broad perspective on the field.

The supplementary search also included a snowball search examining reference lists and citation searches of relevant key articles.

Publication dates were limited to 2010–2022 and searches were limited to English or Danish language.

This review differentiates terminologically between studies and articles throughout the presentation of findings. Studies were defined as peer-reviewed scientific studies identified through the database search. Articles were defined as sources identified in the supplementary search which included grey literature such as news articles etc.

### Defining decision support

This review defines decision support or aid for GPs as the dissemination of patients’ healthcare information to GPs to provide an information base in consultations and patient care, or as the automatic identification of patients at risk of disease. This could, for example, be a digital tool that alerts GPs of high-risk patients, thus informing GP decision-making and possibly enhancing diagnostic accuracy in general practice. To clarify the definition, this review defines digital solutions for decision support as material/tools made available to the GP through electronic software systems and not as analogue material (e.g. on-paper tools).

**Fig. 1:**
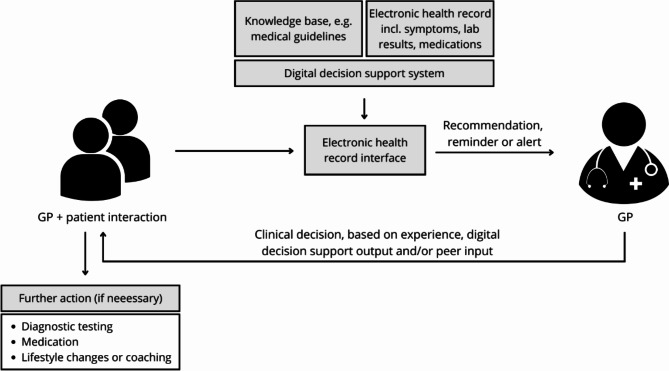
Example of a digital decision support system in a Danish GP setting

Figure 1 below depicts how digital decision support could be used in a Danish GP setting. Depicted is the setting of a consultation between a GP and a patient. The basis of any care decision is clinical experience, medical guidelines, symptoms, lab results, medication information etc. Digital decision support contributes to the GP’s decision-making by generating a reminder, recommendation, alert etc. which the GP may consider in combination with other clinical information in deciding upon any further action for the patient. The decision rests ultimately with the GP.

### Inclusion and exclusion criteria

The inclusion criteria were as follows:

- Digital solution e.g. pop-up, app, etc.
- Decision support or aid for GPs.
- Danish setting.
- General practice setting.
- Published after 2010.
- Peer reviewed (Only applied to database search).

The exclusion criteria were as follows:

- Foreign setting (non-Danish).
- Published before 2010.
- Not peer-reviewed (Only applied to database search, grey literature was included in the supplementary search).
- Hospital setting or other non-GP settings (e.g. municipal health centers).

If the inclusion or exclusion criteria could not be assessed on abstract alone publications were included for full-text screening.

### Risk-of-bias assessment

As this rapid review did not aim to evaluate intervention effects, but only to identify digital solutions developed for or implemented in a Danish general practice setting, a risk-of-bias assessment was deemed unnecessary [10].

### Screening process

Two reviewers, Anne Clausen (AC) and Emilie Rosenfeldt Christensen (ERC) dual-screened 20% of titles and abstracts of studies identified through the database search, with conflict resolution. One reviewer (AC) screened the remaining abstracts and the second reviewer (ERC) screened all excluded abstracts and resolved any conflicts if needed [11]. All articles from the supplementary Infomedia search were dual-screened. The snowball search was conducted independently by both reviewers and findings were discussed until an agreement was reached.

## 3. Results

**Fig. 2:**
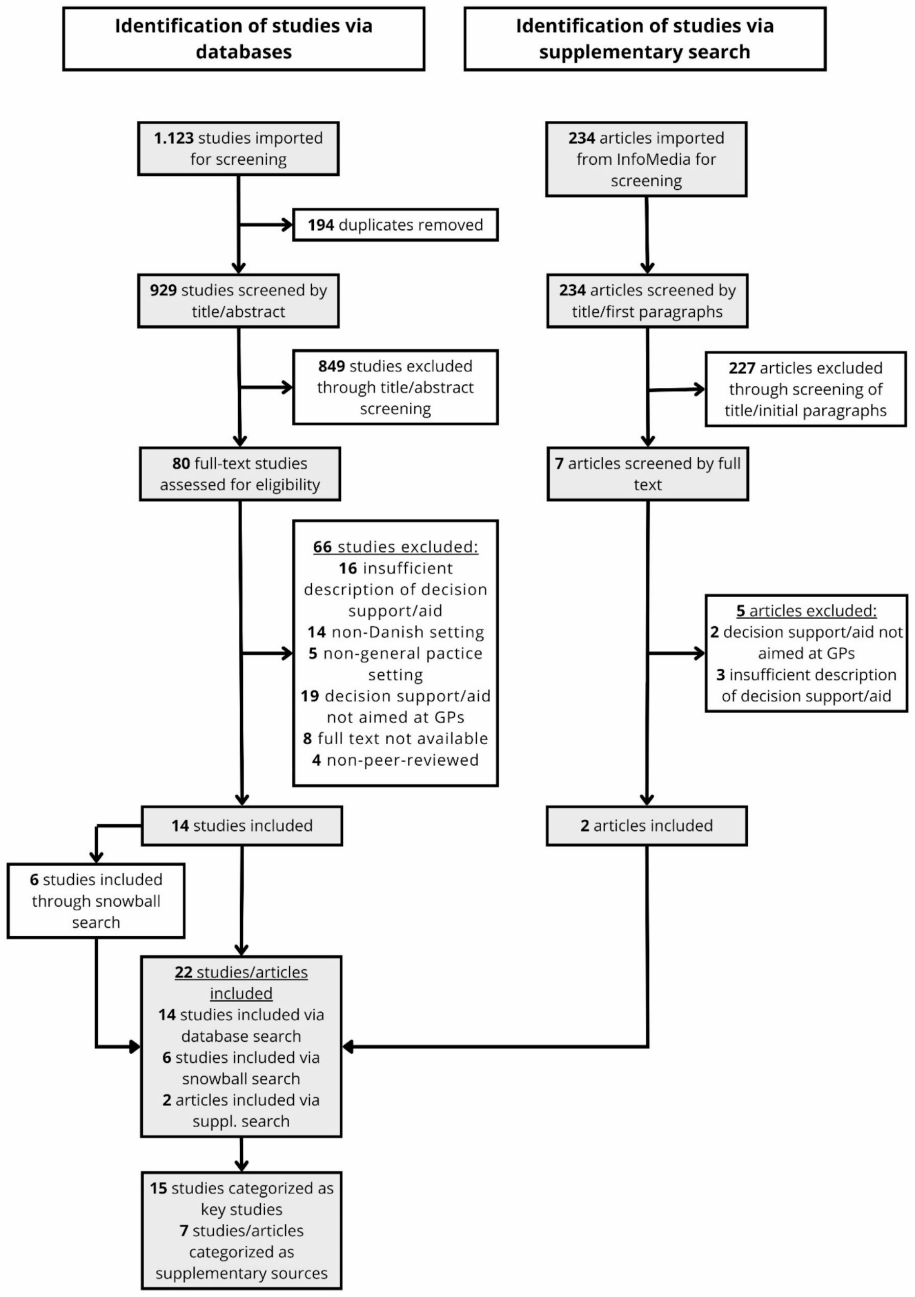
Flow diagram of the screening process

The flow of the screening process is shown in Fig. 2 and described in detail in the following.

A total of 1.123 studies were identified through the database search. After removing 194 duplicates, 929 studies were screened on title and abstract. Through the title and abstract screening, 849 studies were excluded based on the criteria outlined above. If the inclusion and exclusion criteria could not be assessed through the title or abstract alone the study was included for full-text screening for further investigation. A total of 80 studies were screened by full text, of which 66 were excluded based on the following: insufficient description of decision support/aid (n = 16), non-Danish setting (n = 14), non-general practice setting (n = 5), decision support/aid not aimed at GPs (n = 19), full text not available (n = 8), and non-peer-reviewed (n = 4). The remaining 14 studies were included in the review as key articles.

The supplementary search in Infomedia identified 234 articles, which were screened on title and first paragraphs. After the assessment of the title and first paragraphs, 227 articles were excluded based on the criteria outlined above except for the criteria of peer review as grey literature was permitted for inclusion. The full text was retrieved and screened for 7 articles, of which 5 were excluded for the following reasons: decision support/aid not aimed at GPs (n = 2) and insufficient description of decision support/aid (n = 3). The 2 remaining articles described solutions that had already been identified through the primary database search. These 2 articles were therefore included only as supplementary sources for the description of the identified digital solutions.

Through snowball search 6 studies were included. After assessment in full text, one of these studies was included in the review as a key article as it described a digital solution not identified by the primary database search, while the remaining 5 were included as supplementary sources.

A total of 15 studies [12–26] were included in the review as key studies and 7 studies/articles [27–33] were included as supplementary sources for further description of identified digital solutions. The characteristics of the 15 key studies are outlined in Table 1.

**Table 1:** Characteristics of key studies

Author	Study aim	Study population	Participant groups	Targeted condition(s)	Digital solution component(s)	Study period	Outcome measure(s)
Mukai et al. (2012) [12]	To test whether access to existing information can be increased by inserting a hyperlink into electronic test results	n = 300 GPs	1. Standard communication (n = 100) 2. Standard communication and 1 email (n = 100) 3. Standard communication and 2 emails (n = 100)	Breast cancer	Hyperlink / information technology	1 February 2009 to 31 October 2009	Self-reported use of hyperlink
Schroll et al. (2012) [13]	To describe the changes in quality of care in general practice using the program	n = 14.173 patients^a^ / 196 practices	1. Patients with two HbA1c measurements (n = 7.988) 2. Patients with two blood pressure measurements (n = 5.805) 3. Patients with two cholesterol measurements (n = 7.123)	Type 2 Diabetes	Electronic data capture tool and report server	October 2009 to October 2010	Proportion of diabetes cases classified as either controlled or uncontrolled based on different parameters
Smidth et al. (2013) [14]	To test the impact of a model for a Chronic Obstructive Pulmonary Disease (COPD) management program	n = 1.372 patients	1. Intervention group (n = 458) 2. Control group (n = 376) 3. External control group (n = 538)	COPD	Patient identification algorithm	November 2008 to November 2010	Program adherence measured through the use of specific services, as well as the use of out-of-hours services, hospital admissions, etc.
Mukai et al. (2013) [15]	To test whether a web-based clinical decision support system affects PSA testing in general practice	n = 348 practices / 740 GPs	1. Intervention group (n = 114 practices / 247 GPs) 2. Control group (n = 243 practices / 493 GPs)	Prostate cancer	Clinical decision support system	1 January 2010 to 30 Jun 2011	Number of PSA tests (age-standardized) per 1000 men per general practice
Kristiansen et al. (2017) [16]	To test the effect of reminders to GPs regarding missed follow-up after abnormal cervical cytology results	n = 152.551 patients	1. Before group (n = 33.020) 2. Transition group (n = 52.363) 3. After group (n = 60.725)	Cervical cancer	Electronic reminder system	1 January 2009 to 30 May 2014	Proportion of abnormal cervical cytologies without follow-up
Christensen et al. (2018) [17]	To examine the unfolding of the TeleCare North program in three different healthcare settings	n = 15 health professionals	1. Municipal nurses (n = 5) 2. Hospital nurses (n = 2) 3. Lung physicians (n = 2) 4. GPs (n = 6)	COPD	Telemonitoring system	February 2014 to February 2015	Qualitative measures based on interviews, observations, and document studies
Krog et al. (2018) [18]	To identify barriers and facilitating factors to using the web-based tool	n = 9 GPs / 8 practices	N/A	Depression	Telemedicine intervention	February 2017 to April 2017	Qualitative measures based on interview responses
Winthereik et al. (2018) [19]	To develop and conduct pilot testing of an intervention supporting end-of-life care	Unclear^c^	1. CME^d^ meeting attendants (n = 120 GPs) 2. EDS sign-ups (n = 50 GPs)	Cancer and COPD	CME^d^ and clinical decision support system	Spring 2014	Questionnaires, interviews, and emails to gage GP experiences Data regarding EDS^e^ use Patient-related outcomes, e.g. number of terminal declarations, prescriptions, and home deaths
Mønsted (2019) [20]	To examine challenges related to achieving veracity in development and use of a stratification algorithm	n = 13 patients and 5 GPs	N/A	Multiple lifestyle-related diseases^f^	Stratification algorithm	2016	Qualitative measures based on interview responses
Larsen et al. (2019) [21]	To examine attendance in a targeted preventive program and the characteristics of patients who took up the program	n = 2.661 patients	1. Patients diagnosed and/or receiving medical treatment for lifestyle-related disease(n = 699) 2. Patients at high risk of lifestyle-related disease - advised to consult GP (n = 582) 3.Patients engaging in risk behavior - advised to schedule phone-based counseling (n = 618) 4.Patients not exhibiting risk behaviors and not receiving medical treatment (n = 762)	Multiple lifestyle-related diseases^f^	Stratification algorithm and personal health profile	April 2016 to December 2016	Attendance, defined as attending a GP medical examination or telephone-based counselling
Broholm-Jørgensen et al. (2020) [22]	To examine preventive health dialogues from both GP and patient perspectives	n = 11 patients 7 GPs^g^	N/A	Multiple lifestyle-related diseases^f^	Stratification algorithm, digital support system, and personal health profile	2016	Qualitative measures based on interviews and observations
Soerensen et al. (2021) [23]	To develop and validate an AI model to predict 90-day cancer risk based on blood tests	n = 6.592 analytical profiles^h^	1. Development cohort (n = 5.224) 2. Validation cohort (n = 1.368)	Cancer	AI model	29 November 2011 to 1 March 2020	Cancer diagnosis within 90 days of blood test
Jakobsen et al. (2021) [24]	To describe behavior, test feasibility, and identify important factors in digital lifestyle coaching of patients with type 2 Diabetes	n = 15 health professionals / 4 practices	1. Practice nurses (n = 6) 2. GPs (n = 9)	Type 2 Diabetes	Digital lifestyle coaching and treatment	August 2019 to September 2019	Qualitative measures based on interviews
Charles et al. (2022) [25]	To examine whether participation in the program increased the probability of GPs prescribing lipid-lowering medication	n = 9.071 patients and 300 GPs	1. Patients attending one of the 165 ‘exposed’ GPs (n = 5.135) 2. Patients attending one of the 135 ‘control’ ‘GPs (n = 3.936)	Type 2 Diabetes	Electronic disease management program	2011 to 2013	Odds ratio, describing the odds of receiving a prescription for lipid-lowering medication
Blanes-Vidal et al. (2022) [26]	To develop and evaluate AI models capable of predicting significant liver stiffness	n = 3.352 patients	1. Training, validation, and testing data set (n = 3.017) 2. Hold-out dataset (n = 335)	Liver disease	AI models	2013 to 2020	Area Under the Curve, accuracy, sensitivity, specificity, positive predictive value, and negative predictive value

a: Patients may be included in multiple participants groups

b: Pilot testing only. Development phase not included

c: The article does not state how many GPs participated in both the CME^d^ meeting and EDS^e^ system

d: Continued Medical Education

e: Electronic Decision Support

f: Hypertension, Hyperlipidemia, Chronic Obstructive Pulmonary Disease, type 2 Diabetes Mellitus, Cardiovascular disease, and general risk behavior identification

g: Study included 10 observations of preventive health dialogues, 11 interviews with patients, and 7 interviews with GPs. However, it is not stated whether the patients and GPs participating in health dialogues and interviews are the same

h: Blood test profiles, consisting of various laboratory analyses

The 15 key studies described 13 digital solutions for decision support in general practice. The characteristics of the 13 digital solutions are outlined in Table 2. Further elaborations of each digital solution can be found in supplementary material 3.

**Table 2:** Digital solution characteristics

Digital solution	Solution type	Targeted condition(s)	Disease incidence/prevalence in Denmark	Local / national	Purpose	Status
Hyperlink in electronic test communication [12]	Hyperlink / ICT^a^	Breast cancer	4.870 incident cases per year (mean from years 2016–2020) [34]. 74.235 prevalent cases at the end of 2019 [34].	Central Denmark Region	To increase access to existing, patient-oriented information about the disease and its treatment via the web for GPs.	Tested in 148 GP clinics from June to October 2009
Data Capture Module (DCM) for improvement of diabetes care through the National Danish General Practice Database (DAMD) [13]	Pop-ups, diabetes feedback reports on a report server, and access to an online display of data capture	Type 2 diabetes	252.516 prevalent cases as of 2017, equivalent to 4.8% of the Danish population [35]. Approximately 18.700 incident cases per year [35].	Nationwide	To provide GPs access to updated data on the quality of care from their own practice, to identify patients that are not optimally treated.	From April 2011 every Danish GP was obliged within two years to participate in the DCM.
Disease management program (DMP) for Chronic Obstructive Pulmonary Disease (COPD) [14, 27, 28]	DMP including a patient identification algorithm and clinical decision support system	COPD	Prevalence is estimated to 300.000 to 400.000 patients and approximately 14% among individuals aged 35 years and above [36].	Ringkoebing-Skjern Municipality, Denmark	To change/improve the management of COPD in general practice. Effect was measured on planned and additional preventive consultations, performed spirometries, and admissions.	RCT conducted from November 2008 to December 2010
Online decision support system for Prostate-Specific Antigen (PSA) tests [15]	Hyperlink in an electronic medical record system	Prostate cancer	4.542 incident cases per year (mean from years 2016–2020) [37]. 45.610 prevalent cases at the end of 2019 [37].	Central Denmark Region	To aid GPs in deciding if a patient should have a PSA test done, to guide the interpretation of PSA results, or to guide the pathway in a fast-track diagnostic program.	Tested in 114 practices from January 1st 2010 to June 30th 2011
GP reminders on follow-up of abnormal cervical cytology [16]	Electronic GP reminder	Cervical cancer	342 incident cases per year (mean from years 2016–2020) [38]. 8.962 prevalent cases at the end of 2019 [38].	Nationwide	To reduce loss to follow-up in cervical cancer screening	Implemented in 2012
TeleCare North [17, 29]	Telemonitoring patient data made available to GPs through an electronic monitoring database	COPD	Prevalence is estimated to 300.000 to 400.000 patients and approximately 14% among individuals aged 35 years and above [36].	North Denmark Region	To improve the management of COPD patients by providing GPs with telemonitoring data on oxygen level, blood pressure, pulse, weight, and symptoms to support GP decision-making.	Implemented in 2012–2014
Electronic Major Depression Inventory (eMDI) [18]	Through WebPatient the eMDI score is automatically returned to the GP’s electronic patient record upon filling out from patients	Depression	It is estimated that around 10% of the adult Danish population has depression (estimated on 2013 data) [39]. This is equivalent to around 300.000-375.000 prevalent cases [40].	Nationwide	To test psychometric testing of potentially depressive patients in general practice through a telemedicine solution	WebPatient and the possibility to order eMDI testing was implemented nationally in 2015
Electronic Decision Support (EDS) to support end-of-life care [19]	The EDS consisted of a pop-up window in the patient’s medical record and a list of patients with end-of-life needs and key elements in their care	Cancer and COPD	COPD prevalence is estimated to 300.000 to 400.000 patients and approximately 14% among individuals aged 35 years and above [36].	Central Denmark Region	To support end-of-life care in general practice for patients with cancer or COPD	Pilot-tested in the Central Denmark Region in 2014
Early Detection and Prevention (TOF) [20–22, 32, 33]	Patient results from a risk stratification model and a digital data collection tool were made available to GPs through a digital health folder	Multiple lifestyle-related diseases	Not applicable as the definition included a range of lifestyle-related diseases and risk behavior.	Varde and Haderslev municipalities	To develop a health intervention for early detection of citizens at risk of developing lifestyle-related disease and initiation of preventive care	TOF pilot study took place from September 2016 to December 2016
AI to identify patients at risk of cancer [23]	AI risk score to predict cancer within 90 days	Cancer	45.205 incident cancer cases as of 2020 [41]. 362.715 prevalent cases as of 2020 [41].	Region of Southern Denmark	The AI risk score could be a useful tool in decision-making and support GP triage.	Developed on data from the period November 29th 2011 to December 31st 2018. Not yet tested or implemented in general practice.
Digital individualized coaching and lifestyle treatment intervention of T2D (DICTA) [24, 30, 31]	The intervention comprises, among other elements, an algorithm-based decision support for GPs implemented in the GP software system XMO	Type 2 diabetes	252.516 prevalent cases as of 2017, equivalent to 4.8% of the Danish population [35]. Approximately 18.700 incident cases per year [35].	Region of Southern Denmark and Region Zealand	The algorithm-based decision support can support GPs in prescribing the correct medical treatment.	The intervention was developed with GPs and pilot-tested in general practices in the Region of Southern Denmark in 2019. The intervention is rolled out in a RCT running from January 2021 to end in 2023.
Electronic patient data overview with alerts for management of T2D patients [25]	Electronic overview of T2D patients including red flags for patients not receiving treatment as recommended by guidelines.	Type 2 diabetes	252.516 prevalent cases as of 2017, equivalent to 4.8% of the Danish population [35]. Approximately 18.700 incident cases per year [35].	Nationwide	To increase GP prescriptions of lipid-lowering drugs in T2D patients.	The overview was available to Danish GPs in the period from 2011 to 2014.
AI for identification of liver fibrosis patients (LiverAID models) [26]	AI algorithm to predict liver stiffness.	Liver fibrosis	Approximately 1000 incident cases per year of alcoholic liver cirrhosis and an estimated prevalence of 12–14.000 [42]. For non-alcoholic fatty liver disease, there is an estimated prevalence of around 1.000.000 individuals [43].	Region of Southern Denmark	The LiverAID models could be used for the early detection of patients with asymptomatic chronic liver diseases in primary care.	The LiverAID models were developed with data from patients recruited in the period 2013 to 2020. Not yet tested or implemented in general practice.

a: Information and communication technology

## Knowledge summary

This rapid review identified 15 key studies (Table 1) describing 13 digital solutions (Table 2) for decision support in general practice in Denmark. The 13 solutions were aimed at the following disease areas: cancer (n = 5), COPD (n = 3), type 2 diabetes (n = 3), depression (n = 1), liver disease (n = 1), and multiple lifestyle-related diseases (n = 1). Of the 13 solutions, 4 were either developed, tested, or implemented on a national scale and 9 in a limited number of regions or municipalities. As this review did not include an investigation of implementation status beyond what was reported in the identified literature, it was overall not possible to evaluate the current implementation status of the digital solutions as of 2022 (Table 2). Over the course of the inclusion period (2010–2022), there was a progression in the digital solutions as they appeared more complex in recent years. The first study from 2012 described a simple hyperlink solution inserting hyperlinks into electronic test communication [12], whereas the latest study from 2022 described an advanced AI model for the identification of liver disease [26].

### Study strengths & limitations

A methodological strength of this rapid review is the use of dual screening to avoid the subjective bias of using a single reviewer. This was applied in both the primary database search and the supplementary search including the snowball search. The dual-screening reduces the risk of missing key material as two reviewers independently reviewed search materials. The snowball search is a methodological strength as it provides a broad overview of the subject area through citation and reference list searches thereby possibly uncovering relevant material that was not identified through the database search. Further, a strength is the inclusion of feasibility and development studies to achieve a thorough understanding of the included digital solutions and preserve an open scope toward novel approaches. Lastly, researchers of various backgrounds contributed to the generation of this paper, which improved the quality of the final work.

A limitation of this review could be the applied definition of decision support. The definition is relatively broad which possibly means that solutions categorized as decision support for this review may not be categorized as such by others. A methodological limitation is that included studies did not undergo quality assessment which may result in variations of study design, sample size, and outcome measures making it difficult to summarize findings. However, as this rapid review did not aim to evaluate intervention effects, a quality assessment was deemed unnecessary [10]. Furthermore, it can be discussed if the search in MEDLINE and Embase was sufficient or other databases like PubMed should have been included as well. We do not necessarily believe that this is a methodological limitation to this review as the applied health app filters were developed for and validated in these databases [9], and some search techniques are not supported by PubMed. Additionally, the Cochrane Handbook recommends MEDLINE and Embase as search engines for systematic literature searches [44]. However, we do recognize that there might be a difference of opinions about this topic. Another limitation of this rapid review is that it does not include an investigation, beyond the identified literature, of whether the identified digital solutions were ever fully implemented or are still in use as of 2022.

The potential impact of digital solutions in the healthcare system should be considered. The potential impact will necessarily depend on country setting and the organisational structure of the specific healthcare system. In the Danish example, general practice acts as the gateway to the healthcare system, which means that GPs can refer to patient care at hospitals and specialists. In the Danish GP-setting, digital solutions can aid GPs in decision-making in referral for patient care in other segments of the healthcare system by providing a knowledge base or providing prompts/alerts for individuals at potential risk of disease. On one hand, this potential can favour the argument that a strategic goal of improved early detection of disease is realistically achieved as referrals for patient care e.g. at the hospital or at specialist clinics will be supported by an additional knowledge base. On the other hand, it should be considered whether multiple decision support tools could create an information overload that will hamper successful implementation. When implementing digital solutions in the healthcare system it should therefore be carefully considered what the health priorities are in the specific healthcare setting. Continuing the discussion of the potential impact of digital solutions, it should also be addressed whether these tools may contribute to over- or underdiagnosis. Imprecise tools or solutions which do not consider important factors could result in misleading support to healthcare professionals, leading to incorrect or missed diagnosis [45]. Additionally, overreliance on digital solutions may lead to overdiagnosis, as the tools identify patients which would not otherwise have sought medical attention and who do not require treatment [46]. As such, these potential issues underline the need for these tools to be used only as decision support, in conjunction with the GPs own critical assessment.

## Conclusion

In conclusion, this review identified 13 digital solutions for decision support in general practice in a universal healthcare setting in Denmark. The digital solutions covered a range of disease areas (cancer (n = 5), COPD (n = 3), type 2 diabetes (n = 3), depression (n = 1), liver disease (n = 1) and multiple lifestyle-related diseases (n = 1)). Of the 13 solutions, 4 were developed, tested, or implemented on a national scale, and the remaining 9 on a local scale (regional or municipal). The review identified digital solutions with great potential for supporting decision-making in general practice, however, a key learning point is a lack of focus of these studies on how digital solutions are tested, evaluated, and adapted for implementation purposes in general practice. Implementation status could be more transparently reported in publications to enable comparisons across digital solutions and evaluate applicability in general practice. Future studies should consider implementation aspects as part of unfolding the potential of digital solutions as decision support to aid general practitioners in disease detection and management.

### Electronic supplementary material

Below is the link to the electronic supplementary material.

Supplementary Material 1

## Data Availability

Search strings are provided in supplementary material to enable reproducibility of literature searches.

## Abbreviations

COPD: Chronic Obstructive Pulmonary Disease
OECD: Organisation for Economic Co-operation and Development
GP: General Practice
GPs: General Practitioners
PSA: Prostate Specific Antigen
N/A: Not Applicable
CME: Continued Medical Education
EDS: Electronic Decision Support
AI: Artificial Intelligence
ICT: Information and Communication Technology
DCM: Data Capture Module
DMP: Disease Management Programme
RCT: Randomized Controlled Trial
eMDI: Electronic Major Depression Inventory
TOF: Early Detection and Prevention (in Danish:Tidlig Opsporing og Forebyggelse)
T2D: Type 2 Diabetes
