# Supplementary material for: Digital solutions for decision support in general practice – a rapid review focused on systems developed for the universal healthcare setting in Denmark
Source: BMC Prim Care. 2023 Dec 14;24:276. doi: 10.1186/s12875-023-02234-y (PMC10720123; doi:10.1186/s12875-023-02234-y)
Supplement: Supplementary file 1 — Supplementary Material 1 [file 12875_2023_2234_MOESM1_ESM.docx]

## Supplementary material 1 to the manuscript ”Digital solutions for decision support in general practice – a rapid review focused on systems developed for the universal healthcare setting in Denmark”

### PRISMA checklist

| **Section and Topic** | **Item #** | **Checklist item** | **Location where item is reported** |
| --- | --- | --- | --- |
| **TITLE** | | |  |
| Title | 1 | Identify the report as a systematic review. | Page 1. |
| **ABSTRACT** | | |  |
| Abstract | 2 | See the PRISMA 2020 for Abstracts checklist. | Page 3. |
| **INTRODUCTION** | | |  |
| Rationale | 3 | Describe the rationale for the review in the context of existing knowledge. | Page 4. |
| Objectives | 4 | Provide an explicit statement of the objective(s) or question(s) the review addresses. | Page 4. |
| **METHODS** | | |  |
| Eligibility criteria | 5 | Specify the inclusion and exclusion criteria for the review and how studies were grouped for the syntheses. | Pages 6+7. |
| Information sources | 6 | Specify all databases, registers, websites, organisations, reference lists and other sources searched or consulted to identify studies. Specify the date when each source was last searched or consulted. | Page 5+supplemental material pages 4-7. |
| Search strategy | 7 | Present the full search strategies for all databases, registers and websites, including any filters and limits used. | Page 5+supplemental material pages 4-7. |
| Selection process | 8 | Specify the methods used to decide whether a study met the inclusion criteria of the review, including how many reviewers screened each record and each report retrieved, whether they worked independently, and if applicable, details of automation tools used in the process. | Pages 7+8. |
| Data collection process | 9 | Specify the methods used to collect data from reports, including how many reviewers collected data from each report, whether they worked independently, any processes for obtaining or confirming data from study investigators, and if applicable, details of automation tools used in the process. | Pages 7+8. |
| Data items | 10a | List and define all outcomes for which data were sought. Specify whether all results that were compatible with each outcome domain in each study were sought (e.g. for all measures, time points, analyses), and if not, the methods used to decide which results to collect. | Not applicable as this is a rapid review without quantitative data. |
|  | 10b | List and define all other variables for which data were sought (e.g. participant and intervention characteristics, funding sources). Describe any assumptions made about any missing or unclear information. | Not applicable as this is a rapid review without quantitative data. |
| Study risk of bias assessment | 11 | Specify the methods used to assess risk of bias in the included studies, including details of the tool(s) used, how many reviewers assessed each study and whether they worked independently, and if applicable, details of automation tools used in the process. | Page 7. |
| Effect measures | 12 | Specify for each outcome the effect measure(s) (e.g. risk ratio, mean difference) used in the synthesis or presentation of results. | Not applicable as this is a rapid review without quantitative data. |
| Synthesis methods | 13a | Describe the processes used to decide which studies were eligible for each synthesis (e.g. tabulating the study intervention characteristics and comparing against the planned groups for each synthesis (item #5)). | Pages 6-11. |
|  | 13b | Describe any methods required to prepare the data for presentation or synthesis, such as handling of missing summary statistics, or data conversions. | Not applicable as this is a rapid review without quantitative data. |
|  | 13c | Describe any methods used to tabulate or visually display results of individual studies and syntheses. | Not applicable as this is a rapid review without quantitative data. |
|  | 13d | Describe any methods used to synthesize results and provide a rationale for the choice(s). If meta-analysis was performed, describe the model(s), method(s) to identify the presence and extent of statistical heterogeneity, and software package(s) used. | Pages 6-11. |
|  | 13e | Describe any methods used to explore possible causes of heterogeneity among study results (e.g. subgroup analysis, meta-regression). | Not applicable as this is a rapid review without quantitative data. |
|  | 13f | Describe any sensitivity analyses conducted to assess robustness of the synthesized results. | Not applicable as this is a rapid review without quantitative data. |
| Reporting bias assessment | 14 | Describe any methods used to assess risk of bias due to missing results in a synthesis (arising from reporting biases). | Not applicable as this is a rapid review without quantitative data. |
| Certainty assessment | 15 | Describe any methods used to assess certainty (or confidence) in the body of evidence for an outcome. | Not applicable as this is a rapid review without quantitative data. |
| **RESULTS** | | |  |
| Study selection | 16a | Describe the results of the search and selection process, from the number of records identified in the search to the number of studies included in the review, ideally using a flow diagram. | Pages 8-11. |
|  | 16b | Cite studies that might appear to meet the inclusion criteria, but which were excluded, and explain why they were excluded. | Explanations provided on page 10, however citations were not provided. |
| Study characteristics | 17 | Cite each included study and present its characteristics. | Pages 11-13+19-21, and supplemental materials pages 8-11. |
| Risk of bias in studies | 18 | Present assessments of risk of bias for each included study. | Not applicable and explanation for this provided on page 7. |
| Results of individual studies | 19 | For all outcomes, present, for each study: (a) summary statistics for each group (where appropriate) and (b) an effect estimate and its precision (e.g. confidence/credible interval), ideally using structured tables or plots. | Table 1 on pages 19-21, however quantitative data were not included. |
| Results of syntheses | 20a | For each synthesis, briefly summarise the characteristics and risk of bias among contributing studies. | Characteristics were summarised in tables 1-2 on pages 19-24, and supplemental materials pages 8-11. Risk of bias were not assessed – explanation for this was provided on page 7. |
|  | 20b | Present results of all statistical syntheses conducted. If meta-analysis was done, present for each the summary estimate and its precision (e.g. confidence/credible interval) and measures of statistical heterogeneity. If comparing groups, describe the direction of the effect. | No statistical synthesis conducted as this rapid review did not include any quantitative data. |
|  | 20c | Present results of all investigations of possible causes of heterogeneity among study results. | Not applicable as this is a rapid review without quantitative data. |
|  | 20d | Present results of all sensitivity analyses conducted to assess the robustness of the synthesized results. | Not applicable as this is a rapid review without quantitative data. |
| Reporting biases | 21 | Present assessments of risk of bias due to missing results (arising from reporting biases) for each synthesis assessed. | Not applicable as this is a rapid review without quantitative data. |
| Certainty of evidence | 22 | Present assessments of certainty (or confidence) in the body of evidence for each outcome assessed. | Not applicable as this is a rapid review without quantitative data. |
| **DISCUSSION** | | |  |
| Discussion | 23a | Provide a general interpretation of the results in the context of other evidence. | Not applicable as this was a rapid review which aimed to map digital solutions in a Danish context. A knowledge summary of identified digital solutions were provided on page 11. |
|  | 23b | Discuss any limitations of the evidence included in the review. | Pages 11-13. |
|  | 23c | Discuss any limitations of the review processes used. | Pages 11-13. |
|  | 23d | Discuss implications of the results for practice, policy, and future research. | Pages 12-13. |
| **OTHER INFORMATION** | | |  |
| Registration and protocol | 24a | Provide registration information for the review, including register name and registration number, or state that the review was not registered. | Review not registered as this was a rapid review. |
|  | 24b | Indicate where the review protocol can be accessed, or state that a protocol was not prepared. | Protocol not prepared. |
|  | 24c | Describe and explain any amendments to information provided at registration or in the protocol. | Not applicable. |
| Support | 25 | Describe sources of financial or non-financial support for the review, and the role of the funders or sponsors in the review. | Page 14. |
| Competing interests | 26 | Declare any competing interests of review authors. | Page 14. |
| Availability of data, code and other materials | 27 | Report which of the following are publicly available and where they can be found: template data collection forms; data extracted from included studies; data used for all analyses; analytic code; any other materials used in the review. | The search strategy was provided in supplemental materials, which including the validated health app filters. |

*From:*  Page MJ, McKenzie JE, Bossuyt PM, Boutron I, Hoffmann TC, Mulrow CD, et al. The PRISMA 2020 statement: an updated guideline for reporting systematic reviews. BMJ 2021;372:n71. doi: 10.1136/bmj.n71

For more information, visit: <http://www.prisma-statement.org/>

## Supplementary material 2 to the manuscript ”Digital solutions for decision support in general practice – a rapid review focused on systems developed for the universal healthcare setting in Denmark”

### Search strategy for database search in Embase performed on July 6^th^ 2022:

Embase Classic+Embase <1947 to 2022 July 05>

Date of search: 06.07.2022

1 exp Danish citizen/ or exp Denmark/ 60851

2 (Denmark or Danish or Danmark).af. 664546

3 1 or 2 664546

4 exp general practice/ 89243

5 ((General or family) adj3 (practice or medicine)).tw. 93044

6 exp general practitioner/ or exp primary health care/ 287560

7 4 or 5 or 6 388898

8 exp telehealth/ 72817

9 exp computer assisted diagnosis/ 1312439

10 ((Digital or mobile or smart) adj3 health).ti,ab. 12490

11 digital diagnostic*.ti,ab. 153

12 digital care.ti,ab. 152

13 (telehealth or telemedicine or telemonitoring).ti,ab. 33478

14 exp mobile application/ 20560

15 internet/ 117719

16 exp mobile phone/ 40303

17 personal digital assistant/ 1727

18 Medical Informatics Applications/ 22285

19 computer assisted therapy/ 4814

20 (app or apps).ti,ab. 51229

21 (online or internet or web* or digital* or tele* or google).ti. 228787

22 ((online or internet or web* or digital*) adj3 (based or application* or intervention* or program* or therap*)).ab. 96279

23 (phone* or telephone* or smartphone* or cellphone* or smartwatch*).ti. 29835

24 ((phone* or telephone* or smartphone* or cellphone* or smartwatch*) adj3 (based or application* or intervention* or program* or therap*)).ab. 19769

25 (mobile health or mhealth or m-health or ehealth or e-health or emental or e-mental).ti. 8109

26 ((mobile health or mhealth or m-health or ehealth or e-health or emental or e-mental) adj3 (based or application* or intervention* or program* or therap*)).ab. 5352

27 (mobile* adj3 (based or application* or intervention* or device* or technolog*)).ti,ab. 23052

28 (Connected Devices or Smart Devices or Digital Assistant).ti,ab. 1868

29 exp medical informatics/ or exp electronic health record/ or electronic medical record/ or electronic patient record/ 120980

30 ((Health or medical) adj3 (record* or informati* or data)).ti,ab. 425655

31 (digital record* or personalized medicine or personalised medicine or interoperability).ti,ab. 25774

32 exp artificial intelligence/ or exp machine learning/ or exp natural language processing/ 341534

33 ((Artificial or machine or deep or hierarchical or ambient or comput*) adj3 (intelligence or learning)).ti,ab. 117926

34 ((Computer or automated) adj3 reasoning).ti,ab. 184

35 (Knowledge adj3 (acquisition or representation*)).ti,ab. 6407

36 natural language processing.ti,ab. 5981

37 (AI or NLP).ti,ab. 54164

38 exp social media/ 36590

39 (social media or social network or Facebook or twitter or youtube or Instagram or flickr or Linkedin or blog* or on-line communit* or online communit* or wiki* or big data or open data or data mining or cloud or bluetooth or wearable* or wireless technology).ti,ab. 122415

40 (decision adj3 (support or aid)).ti,ab. 40784

41 8 or 10 or 13 or 14 or 15 or 16 or 17 or 18 or 19 or 20 or 21 or 22 or 23 or 24 or 25 or 26 or 27 or 28 or 29 or 30 or 31 or 32 or 33 or 34 or 35 or 36 or 37 or 38 or 39 or 40 1436793

42 3 and 7 and 41 897

43 limit 45 to yr="2010 -Current" 734

### Search strategy for database search in MEDLINE performed on July 6^th^ 2022:

Ovid MEDLINE(R) ALL <1946 to July 05, 2022>

Date of search: 06.07.2022

1 exp Denmark/ 55094

2 (Denmark or Danish or Danmark).af. 470616

3 1 or 2 471688

4 exp General Practice/ or exp Primary Health Care/ or exp General Practitioners/ or exp Family Practice/ 258900

5 exp Telemedicine/ 40976

6 exp Diagnosis, Computer-Assisted/ 86137

7 ((Digital or mobile or smart) adj3 health).ti,ab. 11447

8 digital diagnostic*.ti,ab. 110

9 digital care.ti,ab. 102

10 (telehealth or telemedicine or telemonitoring).ti,ab. 24698

11 Mobile Applications/ 10232

12 exp Internet/ 93084

13 exp Cell Phone/ 20599

14 exp Computers, Handheld/ 11785

15 Medical Informatics Applications/ 2550

16 Therapy, Computer-Assisted/ 6960

17 (app or apps).ti,ab. 37707

18 (online or internet or web* or digital* or tele* or google).ti. 191733

19 ((online or internet or web* or digital*) adj3 (based or application* or intervention* or program* or therap*)).ab. 70629

20 (phone* or telephone* or smartphone* or cellphone* or smartwatch*).ti. 24479

21 ((phone* or telephone* or smartphone* or cellphone* or smartwatch*) adj3 (based or application* or intervention* or program* or therap*)).ab. 14773

22 (mobile health or mhealth or m-health or ehealth or e-health or emental or e-mental).ti. 7337

23 ((mobile health or mhealth or m-health or ehealth or e-health or emental or e-mental) adj3 (based or application* or intervention* or program* or therap*)).ab. 4913

24 (mobile* adj3 (based or application* or intervention* or device* or technolog*)).ti,ab. 18688

25 (Connected Devices or Smart Devices or Digital Assistant).ti,ab. 1623

26 exp Medical Informatics/ or exp Medical Records Systems, Computerized/ 506615

27 ((Health or medical) adj3 (record* or informati* or data)).ti,ab. 278399

28 (digital record* or personalized medicine or personalised medicine or interoperability).ti,ab. 19181

29 exp Artificial Intelligence/ 149885

30 ((Artificial or machine or deep or hierarchical or ambient or comput*) adj3 (intelligence or learning)).ti,ab. 99850

31 ((Computer or automated) adj3 reasoning).ti,ab. 185

32 (Knowledge adj3 (acquisition or representation*)).ti,ab. 5294

33 natural language processing.ti,ab. 4922

34 (AI or NLP).ti,ab. 39306

35 exp Social Media/ 13745

36 (social media or social network or Facebook or twitter or youtube or Instagram or flickr or Linkedin or blog* or on-line communit* or online communit* or wiki* or big data or open data or data mining or cloud or bluetooth or wearable* or wireless technology).ti,ab. 100535

37 (decision adj3 (support or aid)).ti,ab. 30210

38 5 or 7 or 10 or 11 or 12 or 13 or 14 or 15 or 16 or 17 or 18 or 19 or 20 or 21 or 22 or 23 or 24 or 25 or 26 or 27 or 28 or 29 or 30 or 31 or 32 or 33 or 34 or 35 or 36 or 37 1317794

39 3 and 4 and 38 532

40 limit 39 to yr="2010 -Current" 389

## Supplementary material 3 to the manuscript ”Digital solutions for decision support in general practice – a rapid review focused on systems developed for the universal healthcare setting in Denmark”

### Elaborations of identified digital solutions for decision support in general practice

#### Hyperlinks in electronic test results communication

Mukai et al. inserted a hyperlink into the electronic test result communication that GPs participating in a regional systematic breast cancer screening program received (1). The hyperlink provided access to a website with information on the disease and the breast cancer screening program. Approximately half of the participating GPs used the hyperlink, and results suggested that hyperlinks could be a possible strategy for sharing relevant healthcare information with GPs (1).

#### Data Capture Module (DCM) for improvement of diabetes care

Schroll et al. described the use of a DCM in primary care for monitoring and identifying undertreated patients with type 2 diabetes (2). The IT program Sentinel Data Capture collected data on drug prescriptions, diagnoses, laboratory tests, etc. automatically from the GP’s electronic health records and generated quality reports for each practice. The data was stored in the DAMD database and made available to the GPs through quality reports listing patients by various risk parameters. Once a year the GP would be prompted by a pop-up to enter unstructured information regarding care (diet, exercise, smoking, etc.). Based on the quality reports the GPs could rank patients based on the risk parameters and identify patients in need of further care or preventive action (2).

#### Disease management program for Chronic Obstructive Pulmonary Disease (COPD)

Smidth et al. developed a disease management program for COPD which among other elements provided general practitioners with a list of their patients with COPD identified by a COPD algorithm (3). The COPD algorithm identified patients at risk based on administrative data and alerted the GPs on high-risk individuals (4). Further, the program provided decision support for GPs by allowing general practices to draw on a local consultant in lung diseases and made DVDs/podcasts with advice from a specialist accessible to GPs (3). The program was tested in a cluster-randomized controlled trial which proved that the program changed the management of COPD in primary care (5). The number of planned preventive consultations and the number of performed spirometries increased significantly in intervention practices compared with control practices (5).

#### Online decision support system for prostate-specific antigen (PSA) tests

Mukai et al. created a web-based clinical decision support system (CDSS) provided to GPs through a hyperlink in the electronic medical record system (EMR) (6). Entering of the keywords “PSA” or “PROSTATE” into a medical chart in the EMR facilitated an automatic replacement of the keyword with a hyperlink to the CDSS. The CDSS provided the GP with three decision support options. Option number one aimed at supporting the GP in deciding on PSA testing for patients. Option number two supported the GPs interpretation of PSA test results. Option number three provided a guide to the pathway for a fast-track diagnostic hospital program for suspected prostate cancer (6).

#### GP reminders on follow-up of abnormal cervical cytology

Kristiansen et al. evaluated the effect of an electronic GP reminder system on follow-up of abnormal cervical cytology (7). The reminder system was launched nationwide in Denmark in 2011 and was fully operational in 2012. Cervical cytologies are registered in the Danish Pathology Data Bank (DPDB), and for cervical cytologies with a recommendation of follow-up within 3 months an electronic reminder was provided to GPs if a follow-up cytology was not registered in the DPDB 1 month after deadline of the 3 months follow-up period. The proportion of abnormal cervical cytologies lost to follow-up almost halved following the implementation of the reminder system (7).

#### The TeleCare North program

Christensen et al. described TeleCare North, a large-scale telemonitoring program implemented in the North Denmark Region (8). The program included home telemonitoring of COPD patients. COPD patients self-measured their blood pressure, pulse, weight, and oxygen level as well as answered questions about their symptoms (8, 9). Self-measured patient data were uploaded to a shared monitoring database where it was made available to GPs to inform decision-making in the management of COPD patients. The monitoring database activated alarms if measurements surpassed pre-defined thresholds with the aim of alerting GPs of patients in need of preventive action. This provided GPs with the opportunity to timely initiate action by contacting the patient or initiating/adjusting treatment to prevent further exacerbations and hospitalizations (8).

#### Web-based tool for diagnosis and monitoring of patients with depression (eMDI)

Krog et al. investigated factors affecting the use of the electronic Major Depression Inventory (eMDI) in general practice (10). GPs could request the eMDI through the WebPatient system which was launched as a new initiative in 2015 for implementation in general practices in Denmark over a three-year period (10). The eMDI was sent to the patient’s mailbox via an electronic link and after completion the eMDI score was automatically returned to the patient’s electronic patient record where the GP could access it to support decisions of further preventive action (10).

#### Electronic decision support (EDS) to support end-of-life care in general practice

Winthereik et al. developed and pilot-tested an intervention aimed at patients with cancer or COPD to support end-of-life care in general practice (11). The intervention consisted of continuing medical education (CME) meetings and EDS. On a practice level, the EDS generated a list of all patients with palliative needs in each practice. The EDS was integrated into existing electronic records (11).

#### Early detection and prevention (TOF: Tidlig Opsporing og Forebyggelse)

The TOF project aimed at developing a health intervention for the early detection of citizens at risk of developing lifestyle-related diseases (12-15). The intervention consisted of a stratification model and a digital data collection tool. The stratification algorithm automatically stratified citizens into four risk groups: pre-existing diagnosis, high risk, moderate risk, and low risk. High-risk patients were informed via electronic communication of their risk assessment and encouraged to book a health check with their GP. The risk stratification was based on self-reported data on risk behaviours and data from GP systems. Patient data was stored in a digital health folder accessible to both the patient and the GP. The data available in the digital health folder could inform GP decision-making for the initiation of preventive action (12-15).

#### Artificial intelligence (AI) to identify patients at risk of cancer

Soerensen and colleagues developed an AI model to predict a cancer diagnosis within 90 days (16). The model used widely accessible, routine laboratory blood tests to calculate an easy-to-use risk score for the prediction of a cancer diagnosis. The risk score could aid GPs in decision-making regarding whether a patient should be referred for further testing. The model was developed to support early detection of cancer cases (16).

#### Digital individualized coaching and lifestyle treatment intervention of T2D (DICTA)

The DICTA intervention was developed to optimize the course for T2D patients in general practice. The intervention included a lifestyle app aimed at patients and an algorithm-based decision support system aimed at GPs (17). The intervention is currently being tested in a randomized controlled trial (RCT) expected to end in 2023 and a preceding study investigated the feasibility of DICTA in general practice (17, 18). The algorithm-based decision support system was developed to support GPs in the treatment of T2D patients in general practice including support in prescribing the correct medical treatment based on updated clinical guidelines (17, 19).

#### Electronic patient data overview with alerts for the management of T2D patients

Charles and colleagues evaluated the effect of a disease management program (DMP) aimed at T2D management in general practice (20). The DMP generated a list of all patients with T2D in the practice and included an overview of clinical measures, medication, and diabetes consultations. Additionally, an alert aimed at the GP was produced for patients that were not receiving treatment according to recommended guidelines. The overall aim was to improve treatment for T2D patients in general practice (20).

#### AI for identification of liver fibrosis patients (LiverAID)

Blanes-Vidal and colleagues developed a set of AI algorithms (LiverAID models) with the purpose of predicting liver fibrosis (21). The LiverAID models used patient data to predict clinically significant liver stiffness as a marker for liver fibrosis. Data included information on demographics, physical exams, clinical and laboratory parameters, questionnaires, comorbidities, and medications. The authors suggested that the model could be implemented in general practice for the early detection of liver fibrosis patients (21).

### References

1. Mukai TO, Bro F, Fenger-Grøn M, Olesen F, Vedsted P. Use of hyperlinks in electronic test result communication: a survey study in general practice. BMC Medical Informatics and Decision Making. 2012;12(1):114.

2. Schroll H, Christensen RD, Thomsen JL, Andersen M, Friborg S, Søndergaard J. The danish model for improvement of diabetes care in general practice: impact of automated collection and feedback of patient data. Int J Family Med. 2012;2012:208123.

3. Smidth M, Christensen MB, Olesen F, Vedsted P. Developing an active implementation model for a chronic disease management program. Int J Integr Care. 2013;13:e020.

4. Smidth M, Sokolowski I, Kærsvang L, Vedsted P. Developing an algorithm to identify people with Chronic Obstructive Pulmonary Disease (COPD) using administrative data. BMC Medical Informatics and Decision Making. 2012;12(1):38.

5. Smidth M, Christensen MB, Fenger-Grøn M, Olesen F, Vedsted P. The effect of an active implementation of a disease management programme for chronic obstructive pulmonary disease on healthcare utilization--a cluster-randomised controlled trial. BMC Health Serv Res. 2013;13:385.

6. Mukai TO, Bro F, Olesen F, Vedsted P. To test or not: a registry-based observational study of an online decision support for prostate-specific antigen tests. Int J Med Inform. 2013;82(10):973-9.

7. Kristiansen BK, Andersen B, Bro F, Svanholm H, Vedsted P. Impact of GP reminders on follow-up of abnormal cervical cytology: a before-after study in Danish general practice. Br J Gen Pract. 2017;67(661):e580-e7.

8. Christensen JKB. The Emergence and Unfolding of Telemonitoring Practices in Different Healthcare Organizations. Int J Environ Res Public Health. 2018;15(1).

9. Jungersen DR. 4500 nordjyske KOL-patienter tilbydes behandling i eget hjem. Ugeskriftetdk. 2012.

10. Krog MD, Nielsen MG, Le JV, Bro F, Christensen KS, Mygind A. Barriers and facilitators to using a web-based tool for diagnosis and monitoring of patients with depression: a qualitative study among Danish general practitioners. BMC Health Serv Res. 2018;18(1):503.

11. Winthereik AK, Neergaard MA, Jensen AB, Vedsted P. Development, modelling, and pilot testing of a complex intervention to support end-of-life care provided by Danish general practitioners. BMC Fam Pract. 2018;19(1):91.

12. Mønsted T. Achieving veracity: A study of the development and use of an information system for data analysis in preventive healthcare. Health Informatics Journal. 2018;25(3):491-9.

13. Larsen LB, Sondergaard J, Thomsen JL, Halling A, Sønderlund AL, Christensen JR, et al. Step-wise approach to prevention of chronic diseases in the Danish primary care sector with the use of a personal digital health profile and targeted follow-up – an assessment of attendance. BMC Public Health. 2019;19(1):1092.

14. Broholm-Jørgensen M, Langkilde SM, Tjørnhøj-Thomsen T, Pedersen PV. 'Motivational work': a qualitative study of preventive health dialogues in general practice. BMC Fam Pract. 2020;21(1):185.

15. Thilsing T, Sonderlund AL, Sondergaard J, Svensson NH, Christensen JR, Thomsen JL, et al. Changes in Health-Risk Behavior, Body Mass Index, Mental Well-Being, and Risk Status Following Participation in a Stepwise Web-Based and Face-to-Face Intervention for Prevention of Lifestyle-Related Diseases: Nonrandomized Follow-Up Cohort Study. JMIR Public Health Surveill. 2020;6(3):e16083.

16. Soerensen PD, Christensen H, Gray Worsoe Laursen S, Hardahl C, Brandslund I, Madsen JS. Using artificial intelligence in a primary care setting to identify patients at risk for cancer: a risk prediction model based on routine laboratory tests. Clin Chem Lab Med. 2021.

17. Jakobsen PR. Digital coaching af patienter med type 2-diabetes og klinisk beslutningsstøtte til almen praksis (DICTA). Månedsskrift for almen praksis. 2021.

18. Jakobsen PR, Christensen JR, Nielsen JB, Søndergaard J, Ejg Jarbøl D, Olsen MH, et al. Identification of Important Factors Affecting Use of Digital Individualised Coaching and Treatment of Type 2 Diabetes in General Practice: A Qualitative Feasibility Study. Int J Environ Res Public Health. 2021;18(8).

19. Sjøgren K. Digital løsning skal lette praktiserende lægers arbejde med diabetespatienter. Dagens Medicin. 2021.

20. Charles MH, Thomsen JL, Christensen B, Pulleyblank R, Kongstad LP, Olsen KR. Use of electronic patient data overview with alerts in primary care increases prescribing of lipid-lowering medications in patients with type 2 diabetes. Diabetologia. 2022;65(2):286-90.

21. Blanes-Vidal V, Lindvig KP, Thiele M, Nadimi ES, Krag A. Artificial intelligence outperforms standard blood-based scores in identifying liver fibrosis patients in primary care. Sci Rep. 2022;12(1):2914.
